# Risk factor identification and prediction models for prolonged length of stay in hospital after acute ischemic stroke using artificial neural networks

**DOI:** 10.3389/fneur.2023.1085178

**Published:** 2023-02-09

**Authors:** Cheng-Chang Yang, Oluwaseun Adebayo Bamodu, Lung Chan, Jia-Hung Chen, Chien-Tai Hong, Yi-Ting Huang, Chen-Chih Chung

**Affiliations:** ^1^Department of Neurology, Shuang Ho Hospital, Taipei Medical University, New Taipei City, Taiwan; ^2^Research Center for Brain and Consciousness, Taipei Medical University, Taipei, Taiwan; ^3^Department of Medical Research and Education, Shuang Ho Hospital, Taipei Medical University, New Taipei City, Taiwan; ^4^Department of Urology, Shuang Ho Hospital, Taipei Medical University, New Taipei City, Taiwan; ^5^Department of Hematology and Oncology, Shuang Ho Hospital, Taipei Medical University, New Taipei City, Taiwan; ^6^Department of Neurology, School of Medicine, College of Medicine, Taipei Medical University, Taipei, Taiwan; ^7^Department of Nursing, School of Nursing, College of Medicine, National Taiwan University, Taipei, Taiwan

**Keywords:** artificial neural network - ANN, hospitalization, ischemic stroke, length of stay, machine learning, prediction, thrombolysis, outcome

## Abstract

**Background:**

Accurate estimation of prolonged length of hospital stay after acute ischemic stroke provides crucial information on medical expenditure and subsequent disposition. This study used artificial neural networks to identify risk factors and build prediction models for a prolonged length of stay based on parameters at the time of hospitalization.

**Methods:**

We retrieved the medical records of patients who received acute ischemic stroke diagnoses and were treated at a stroke center between January 2016 and June 2020, and a retrospective analysis of these data was performed. Prolonged length of stay was defined as a hospital stay longer than the median number of days. We applied artificial neural networks to derive prediction models using parameters associated with the length of stay that was collected at admission, and a sensitivity analysis was performed to assess the effect of each predictor. We applied 5-fold cross-validation and used the validation set to evaluate the classification performance of the artificial neural network models.

**Results:**

Overall, 2,240 patients were enrolled in this study. The median length of hospital stay was 9 days. A total of 1,101 patients (49.2%) had a prolonged hospital stay. A prolonged length of stay is associated with worse neurological outcomes at discharge. Univariate analysis identified 14 baseline parameters associated with prolonged length of stay, and with these parameters as input, the artificial neural network model achieved training and validation areas under the curve of 0.808 and 0.788, respectively. The mean accuracy, sensitivity, specificity, positive predictive value, and negative predictive value of prediction models were 74.5, 74.9, 74.2, 75.2, and 73.9%, respectively. The key factors associated with prolonged length of stay were National Institutes of Health Stroke Scale scores at admission, atrial fibrillation, receiving thrombolytic therapy, history of hypertension, diabetes, and previous stroke.

**Conclusion:**

The artificial neural network model achieved adequate discriminative power for predicting prolonged length of stay after acute ischemic stroke and identified crucial factors associated with a prolonged hospital stay. The proposed model can assist in clinically assessing the risk of prolonged hospitalization, informing decision-making, and developing individualized medical care plans for patients with acute ischemic stroke.

## 1. Introduction

Stroke is the second leading cause of death and a significant cause of disability worldwide (1, 2). Stroke results in substantial health and economic burdens on patients and the healthcare system (1–3). Globally, ~12.2 million stroke events are estimated to occur annually (1, 3, 4). With 101 million stroke cases and 6.55 million deaths in 2019 alone, the clinical and economic burdens of stroke on the healthcare system, society, families, and patients are enormous (1, 3, 4). The economic burden includes direct costs of medical and non-medical care and indirect costs such as economic losses due to the loss of productivity (1, 3, 4). The annual cost of stroke management is ~ €60 billion in Europe (4, 5) and US$65.5 billion (3, 6). With the aging and an increase in the number of the population, the number of stroke events and their long-term sequelae and associated costs is projected to increase significantly (3–6). Therefore, identifying the risk factors and predicting the stroke burden are valuable for planning and organizing stroke services.

Depending on stroke severity and its consequences, patients may have a physical disability and require short- or long-term hospital care and rehabilitation or constant care for the rest of their life. Approximately 80% of stroke survivors exhibit motor impairment, 40% develop moderate to severe impairments and require special care, and 10% require long-term care at stroke facilities (3, 7). As one of the factors contributing to total hospitalization costs, prolonged length of stay (LOS) is highly predictive of inpatient costs (8–13). As LOS is a primary determinant of the cost of stroke care and poststroke rehabilitation, it is essential to identify independent predictors of LOS to improve resource allocation and cost efficiency (14).

In addition to hospitalization costs, prolonged LOS may be associated with the increased use of healthcare resources, negatively affecting hospital capacity and medical personnel availability, reducing the quality of care, and preventing other patients from receiving inpatient care (15, 16). Furthermore, patients with acute ischemic stroke (AIS) having prolonged LOS are more likely to develop complications such as hospital-acquired infections or gastrointestinal bleeding (17, 18). Prolonged LOS is also associated with poststroke depression and increased disability, negatively affecting patients' quality of life (19, 20). Prolonged LOS after AIS was also reported to be associated with less favorable neurological outcomes at hospital discharge (17). Therefore, acquiring knowledge of the factors associated with LOS and estimating the likelihood of prolonged LOS enable the prediction of recovery from an AIS episode and are crucial for clinical applications.

Several previously documented models using linear regression or scoring systems for predicting LOS for patients with AIS exhibited limited discrimination (21–23). Conventional linear regression models examine only the direct effects between dependent and independent variables and have limitations in analyzing data with skewed distributions. Linear regression models may also neglect the effect of covariates having multicollinearity with the dependent variable (24–28). Therefore, predicting LOS using a single regression model is difficult and usually ineffective.

An artificial neural network (ANN) is a machine-learning system that simulates the human nervous system (29–31). It assimilates the complexity of input–output non-linear interactions by repeating training and validation processes to optimize inner unit connections and subsequently uses that knowledge to achieve the desired level of prediction accuracy for unobserved situations (29–31). ANN has the advantage of analyzing the complex, multidimensional, or non-linear relationship between variables and outcomes and continues to be widely applied in medical diagnosis, outcome prediction, and healthcare decision-making (25–28, 31, 32).

This study explored the association between neurological outcomes after AIS and LOS, established and validated an ANN-based model to predict prolonged LOS in hospitalized patients with AIS based on clinical parameters obtained at admission, and identified crucial predictors contributing to LOS.

## 2. Materials and methods

### 2.1. Ethics approval

The Joint Institutional Review Board of Taipei Medical University (TMU-JIRB Approval No. N202103006) approved this study. For this retrospective study involving the secondary analysis of existing anonymized data, TMU-JIRB waived the requirement for informed consent. All experiments were performed in accordance with relevant named guidelines and regulations.

### 2.2. Source of data

This study retrieved the medical records of patients who received stoke diagnoses and was treated at Taipei Medical University-Shuang Ho Hospital between January 2016 and June 2020 from the Taiwan Stroke Registry (TSR, http://taiwanstrokeregistry.org/TSR/), and retrospective analysis of the data was conducted. TSR is a multicenter database of the clinical data of patients with stroke admitted to major medical institutions in Taiwan (33).

### 2.3. Participants

The inclusion criteria were as follows: patients who (1) were 18 years or older, (2) received AIS diagnoses and were admitted and treated for AIS at our hospital, and (3) presented to the hospital within 10 days of AIS symptom onset (33). The exclusion criteria were as follows: patients who presented with acute intracranial hemorrhage, without a determined National Institutes of Health Stroke Scale (NIHSS) score on admission, or with incomplete registration information. All patients received non-contrast head computed tomography (CT) or brain magnetic resonance imaging (MRI) on admission. Two independent neurologists and a radiologist interpreted all CT/MRI images. Demographic data, including age, sex, and presence of cerebrovascular risk factors such as hypertension (HTN), diabetes mellitus (DM), hyperlipidemia, ischemic heart disease, atrial fibrillation (Af), and previous stroke, were collected at the presentation. Thrombolytic treatments for AIS, namely, intravenous thrombolysis (IVT) with or without endovascular thrombectomy (EVT) and non-thrombolysis, were also documented. Certified stroke specialists used the NIHSS to assess the overall severity of AIS. Blood cell counts, prothrombin time (PT), and activated partial thromboplastin time were measured at admission. Albumin, fasting glucose, glycated hemoglobin, triglyceride (TG), and low-density lipoprotein cholesterol (LDL) levels were measured within 72 h of access.

### 2.4. Outcome

The LOS of each patient was defined as the time from admission to discharge from the hospital. Prolonged LOS was defined as the length of a hospital stay beyond the median LOS (34, 35).

### 2.5. Statistical analysis

All statistical analyses were conducted using Statistica version 13.3 (TIBCO Software Inc., Tulsa, Oklahoma, USA). Variables were summarized using descriptive statistics. Continuous variables are presented as medians (interquartile ranges [IQRs]), and categorical variables are expressed as counts and proportions (%).

Student's *t*-test was used to compare means between two groups of continuous variables. One-way ANOVA was used for comparing the means of ≥3 independent variables from the prolonged LOS and non-prolonged LOS groups. Pearson's chi-squared test was used to determine non-random associations between two categorical variables from the prolonged and non-prolonged LOS groups. All hypothesis tests were two-sided, with a *p*-value of 0.05 indicating significance.

### 2.6. Development of ANN models

**Figure 1:**
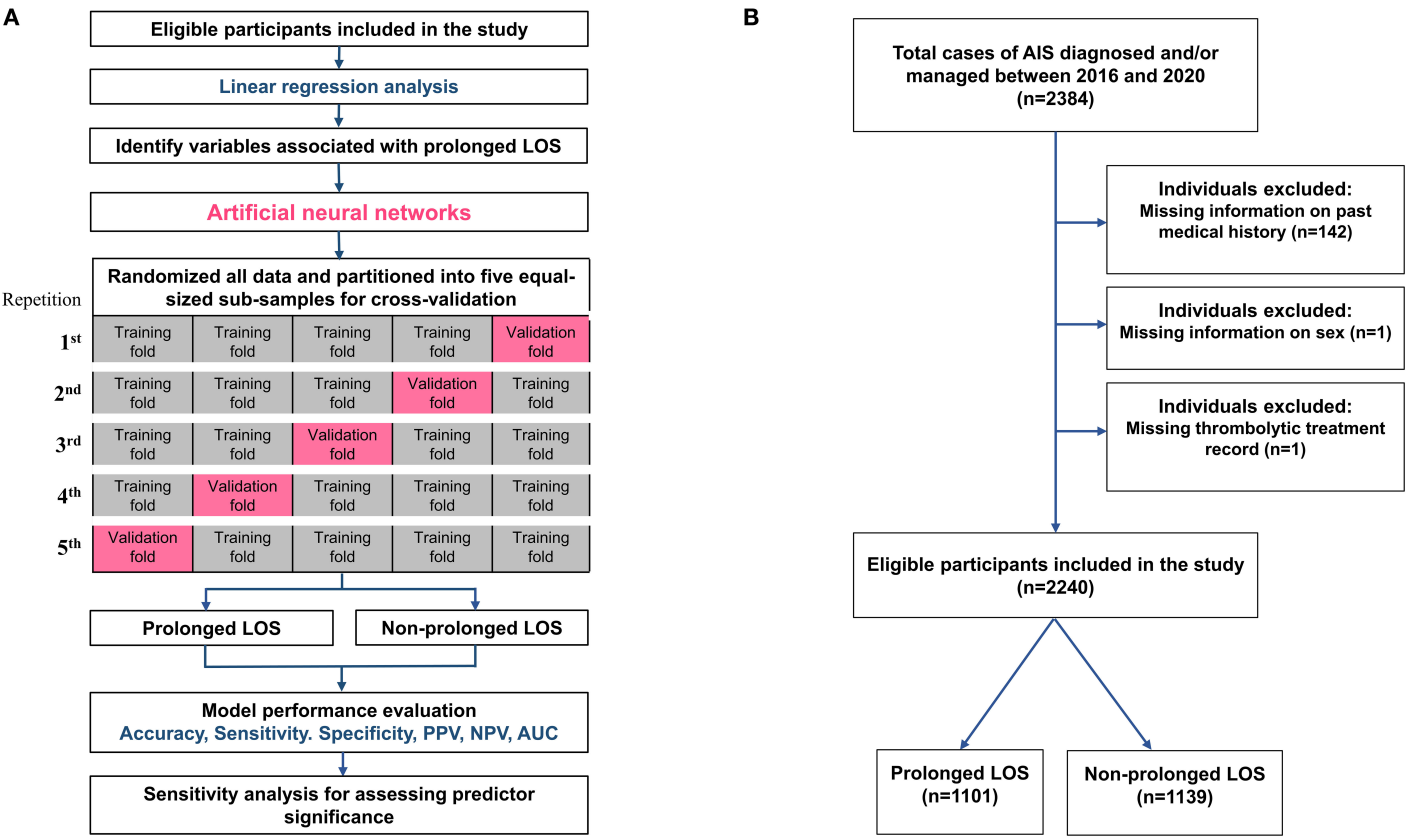
The flow diagram of the study. **(A)** The flow diagram of the development of the current predicting models and the schematic representation of 5-fold cross-validation. **(B)** Flowchart showing the study cohort selection and division based on LOS status. AIS, acute ischemic stroke; AUC, area under the receiver operating characteristic curve; LOS, length of stay; NPV, negative predictive value; PPV, positive predictive value.

The ANN applied in this study was a feedforward neural network of multilayer perceptrons with an input layer, a hidden layer, and an output layer. The ANN model used the standard backpropagation gradient estimation algorithm with a linear combination of input variables within the hidden layer that contained an intercept (bias) and the coefficient for each predictor. The neural networks were trained using the Broyden–Fletcher–Goldfarb–Shanno (BFGS) method (36). The variables used in the input layer included those associated with prolonged LOS in the study cohort (*p* < 0.05 by descriptive statistics, Table 1). Figure 1A shows the flow diagram for the development of the predicting models. Continuous variables included age, NIHSS score at admission, albumin level, fasting glucose level, hemoglobin level, white blood cell (WBC) count, PT, cholesterol level, and TG level. Categorical variables included initial thrombolytic treatments, Af, DM, HTN, and previous stroke. Categorical variables were inputted as neurons and converted using one-hot encoding. During the model training process, the search for optimal hyperparameters was performed with the following limits: 1 hidden layer, 1–50 neurons in the hidden layer, and the activation function was chosen to be either the exponential or the hyperbolic tangent function. The output layer contained two neurons, namely, “prolonged LOS” and “non-prolonged LOS.” The sigmoid activation function was used for the neurons in the output layer. All networks were trained using early stopping. We presented the network with an input-target pair from the training set and computed the predictions of the network for the targets. We used the cross-entropy error function to calculate the difference between the predictions of the network and the target values until all input-target pairs from the training set were presented to the network. The training algorithm was used to adjust the weights of the networks. The error of each training is compared with the error of the previous iteration. Training is continued if the error keeps decreasing; otherwise, training is stopped. All ANN models were developed using Statistica version 13.3 (TIBCO Software Inc.).

**Table 1:** Baseline demographic characteristics of patients according to prolonged length of stay (LOS) at hospital.

**Variables**	**Whole cohort**	**Prolonged LOS**	**Non-prolonged LOS**	***p*-value**
Number of patients	2240	1101	1139	
LOS (days)	9 (6–20)	20 (13–27)	6 (4–7)	
Age (years)	68 (59–79)	70 (61–81)	66 (58.5–77)	< 0.0001^*^
Female, *n* (%)	844 (37.7)	436 (39.6)	408 (35.8)	0.067
NIHSS score at admission	4 (2–10)	7 (4–16)	3 (1–5)	< 0.0001^*^
**Thrombolytic treatment**, ***n*** **(%)**				<0.0001^*^
Non-thrombolysis	1934 (86.3)	899 (81.7)	1035 (90.9)	
IVT	197 (8.8)	120 (10.9)	77 (6.8)	
EVT	109 (4.9)	82 (7.5)	27 (2.4)	
**Laboratory data**				
Albumin, mg/dL	4.0 (3.7–4.2)	3.9 (3.6–4.2)	4.0 (3.8–4.3)	< 0.0001^*^
Fasting glucose, mg/dL	115 (99–149)	122 (102.25–159)	108 (97–137)	< 0.0001^*^
Glycated hemoglobin, %	6.0 (5.6–7.1)	6.0 (5.6–7.2)	6.0 (5.6–7.1)	0.23
Hemoglobin, g/dL	14.1 (12.8–15.3)	14.0 (12.5–15.2)	14.3 (13–15.3)	< 0.0001^*^
White blood cell count, × 10^3^/μL	8.1 (6.5–10)	8.4 (6.8–10.5)	7.8 (6.3–9.6)	< 0.0001^*^
Platelets, × 10^3^/μL	215 (174–261)	215 (175–263)	215 (174–258)	0.83
Prothrombin time, s	12.9 (12.4–13.5)	13 (12.5–13.7)	12.9 (12.3–13.4)	< 0.0001^*^
Activated partial thromboplastin time, s	36.1 (33.3–39.2)	36.1 (33.1–39.2)	36.1 (33.5–39.1)	0.55
Creatinine, mg/dL	0.95 (0.77–1.2)	0.96 (0.77–1.26)	0.93 (0.77–1.15)	0.13
Cholesterol, mg/dL	184 (156–214)	182 (152–214)	185 (159–214)	0.0178^*^
Triglyceride, mg/dL	112 (77–160)	107 (72.5–155)	117.5 (81–166.25)	0.0009^*^
Low-density lipoprotein, mg/dL	112 (89–140)	110 (86–139)	114 (91–140)	0.31
**Vascular risk factors**, ***n*** **(%)**				
Atrial fibrillation	399 (17.8)	248 (22.5)	151 (13.3)	< 0.0001^*^
Diabetes mellitus	871 (38.9)	459 (41.7)	412 (36.2)	0.0082^*^
Hypertension	1572 (70.2)	797 (72.4)	775 (68.0)	0.0266^*^
Previous stroke	348 (15.5)	198 (18.0)	150 (13.2)	0.002^*^
Ischemic heart disease	240 (10.7)	123 (11.2)	117 (10.3)	0.50
Uremia	26 (1.2)	13 (1.2)	13 (1.1)	1.0

Continuous variables were presented as median (interquartile range, IQR), and categorical variables were expressed as counts and proportions (%). *p*-value = comparison of the prolonged and non-prolonged LOS groups. EVT, endovascular thrombectomy; IVT, intravenous thrombolysis; LOS: length of stay; NIHSS, National Institutes of Health Stroke Scale.

* *p* < 0.05 and the variables were used in the input layer of the artificial neural network model.

### 2.7. Model evaluation

To avoid overfitting in the ANN model, we performed 5-fold cross-validation to assess the generalizability of the analysis results. The original sample was randomly partitioned into five equal-sized subsamples. In each repetition of the cross-validation process, one subsample was retained as the validation set, and the remaining four subsamples were used as the training set. The cross-validation process was repeated five times, and each of the five subsamples was used once as the validation set (Figure 1A). Model performance was evaluated based on receiver operating characteristic (ROC) curve analysis, and the area under the ROC curve (AUC) of the five training and validation sets was calculated to represent the level of discrimination. We chose the model with the highest AUC of the cross-validations for further analysis. After the models were developed, their mean accuracy, sensitivity, specificity, positive predictive value (PPV), and negative predictive value (NPV) were derived from the confusion matrix of the five validation sets (Figure 1A).

### 2.8. Relative significance of predictors

To understand the contribution of each predictor to the likelihood of extending LOS after AIS, we estimated the relative importance of each variable in the model as representative of that parameter's relative contribution to the ANN model. We performed a sensitivity analysis of each repetition of the 5-fold cross-validation and reported the mean values (37, 38).

## 3. Results

### 3.1. Study cohort demographics and baseline characteristics

Of the total 2,384 patients diagnosed with AIS and treated at Shuang Ho Hospital during the study period, 144 patients were excluded from baseline analyses because of incomplete registration information (142 with missing data on medical history, 1 with missing thrombolytic treatment records, and 1 without sex information). Consequently, 2,240 patients (844 women and 1,396 men) were included in the analysis (Figure 1B). The median age of the study cohort was 68 years, with an interquartile range (IQR) of 50–79 years. The median baseline NIHSS score at admission was 4 (IQR: 2–10) for the entire cohort. The median NIHSS scores at admission for patients who received non-thrombolytic treatment, IVT, and EVT were 4 (IQR: 2–8), 10 (IQR: 5–17), and 19 (IQR: 14.5–24) (*p* < 0.0001). The mean and median LOS of the entire cohort were 13.6 ± 11.3 and 9 days, respectively, with an IQR of 6–20 days (Table 1). Therefore, a prolonged LOS was defined as a length of stay of more than 9 days. Among patients, 1,101 (49.2%) had prolonged LOSs, with a median hospital stay of 20 days (IQR: 13–27).

Prolonged LOS was associated with different thrombolytic therapies. The median LOS of patients who received non-thrombolytic treatment, IVT, and EVT was 9 (IQR: 6–18), 13 (IQR: 7–25), and 20 (IQR: 9.5–28) days, respectively (*p* < 0.0001). Compared with patients without prolonged LOS, more patients with prolonged LOS were treated with IVT or EVT. Compared with patients without prolonged LOS, patients with prolonged LOS were older; had higher NIHSS scores at admission; had lower albumin, hemoglobin, serum cholesterol, and TG levels; were more likely to exhibit higher fasting glucose levels and WBC counts; and had longer PT upon hospitalization. Patients with prolonged LOS also had a higher prevalence of Af, DM, HTN, and previous stroke (Table 1).

### 3.2. Prolonged LOS and AIS outcomes at hospital discharge

**Figure 2:**
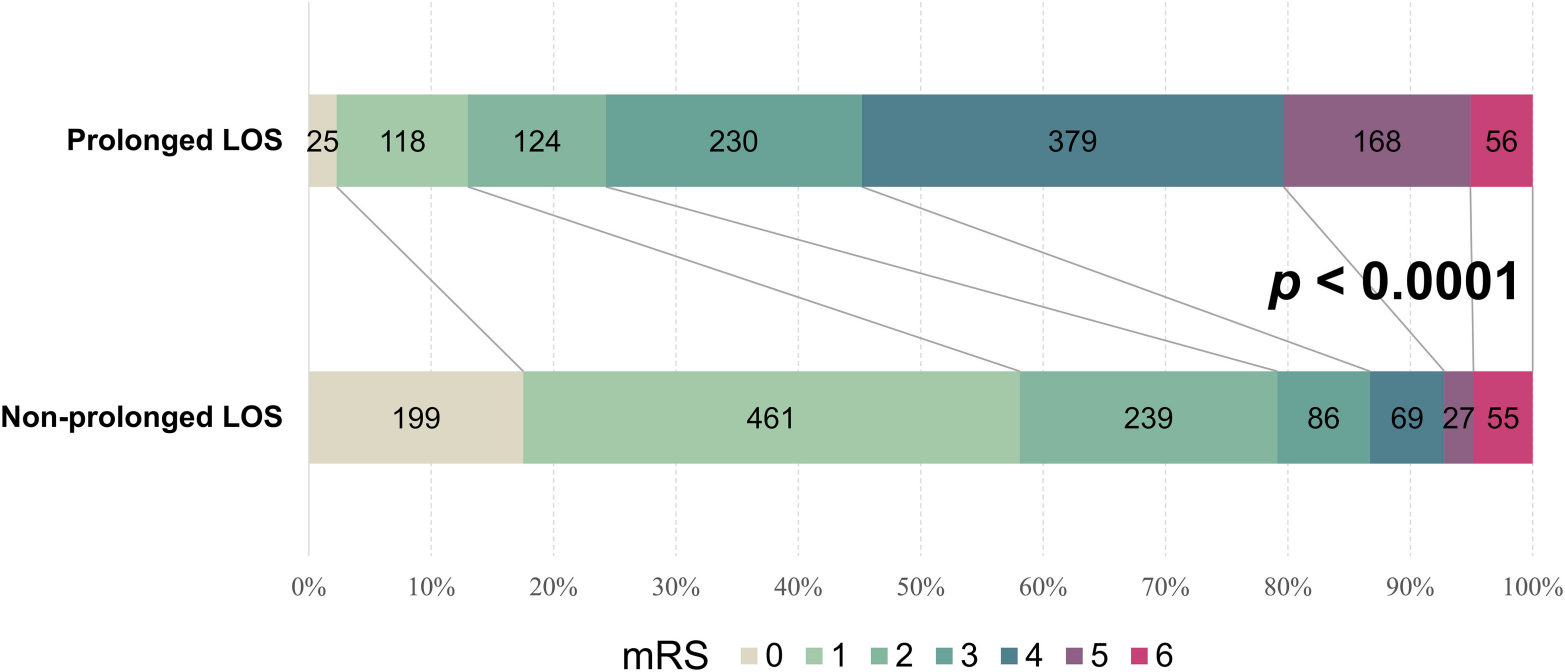
Prolonged LOS-stratified mRS banding of patients with AIS. Graphical representation of the differential mRS of AIS patients with a prolonged or non-prolonged LOS at hospital discharge. Digits in each color-coded bar represent the number of patients with indicated mRS scores. AIS, acute ischemic stroke; LOS, length of stay; mRS, modified Rankin Scale.

We examined associations between prolonged LOS and neurological outcomes at discharge in patients with AIS. In the entire cohort, there were 1,368 patients (61.1%) who had unfavorable neurological outcomes, defined as a modified Rankin Scale (mRS) ≥ 2 at discharge. A higher proportion of the patients with prolonged LOS had unfavorable outcomes than did those with a shorter LOS (87.0 vs. 41.7%, odds ratio [OR] = 9.33; 95% confidence interval [CI] = 7.52–11.59; *p* < 0.0001). Univariable analysis showed that each day's increase in LOS was associated with a 1.2-fold increase in the risk of unfavorable AIS outcomes (95% CI = 1.18–1.22; *p* < 0.0001). For the AIS patients with and without a prolonged LOS, the median mRS at discharge was 4 (IQR: 3–4) and 1 (IQR: 1–2), respectively (*p* < 0.0001). As illustrated in Figure 2, the patients with a prolonged hospital LOS had a higher mRS score at hospital discharge than those without a prolonged LOS, indicating that a prolonged LOS is associated with less favorable outcomes in patients with AIS.

### 3.3. Prolonged LOS prediction using ANNs

**Figure 3:**
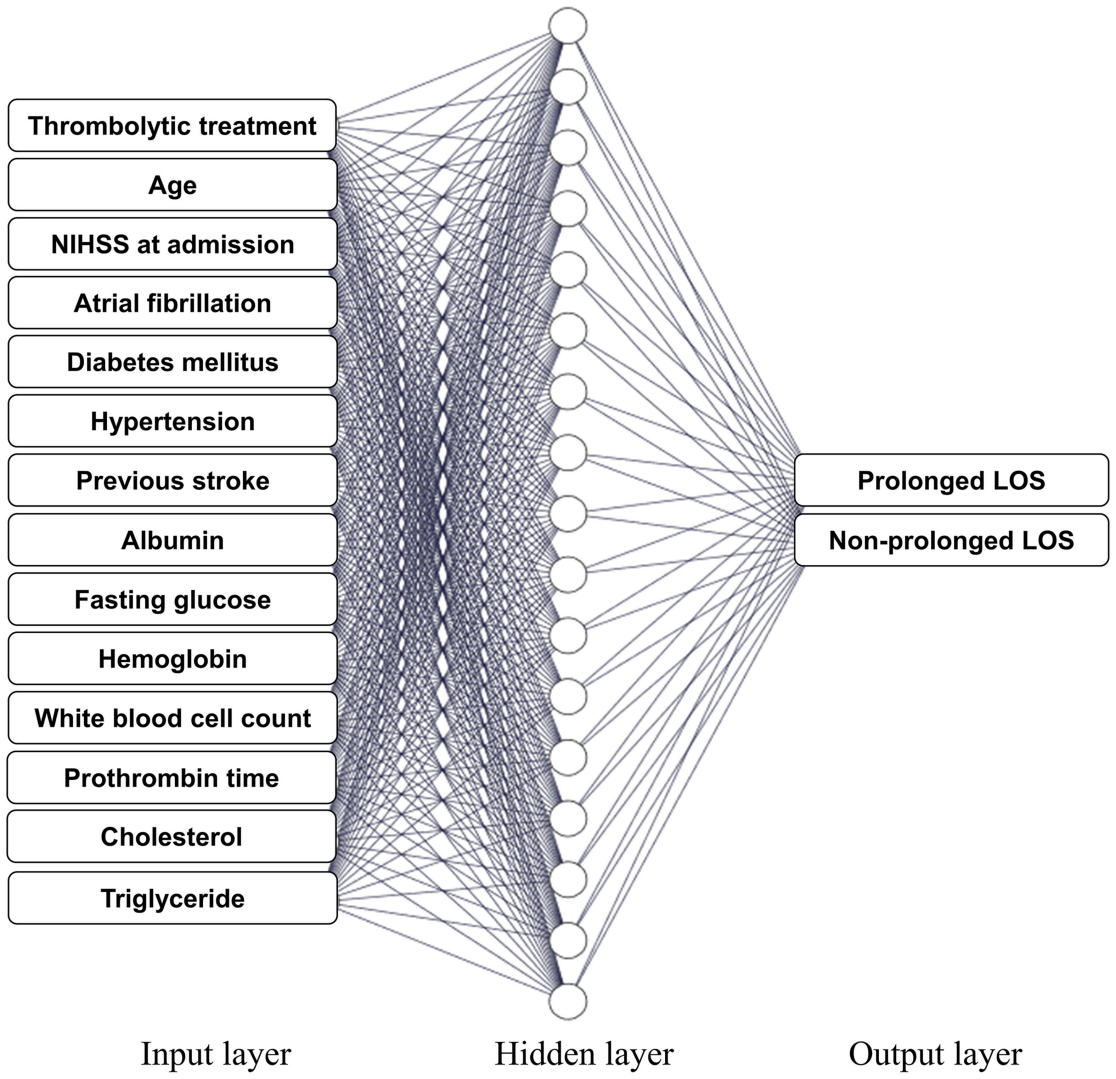
Artificial neural network (ANN) models. Schema of the network structure of the study ANN model consisting of 14 baseline predictor characteristics as the input layer, a hidden layer, and an output layer containing two neurons, namely, prolonged LOS and non-prolonged LOS. ANN, artificial neural network; LOS, length of stay; NIHSS, National Institutes of Health Stroke Scale.

**Figure 4:**
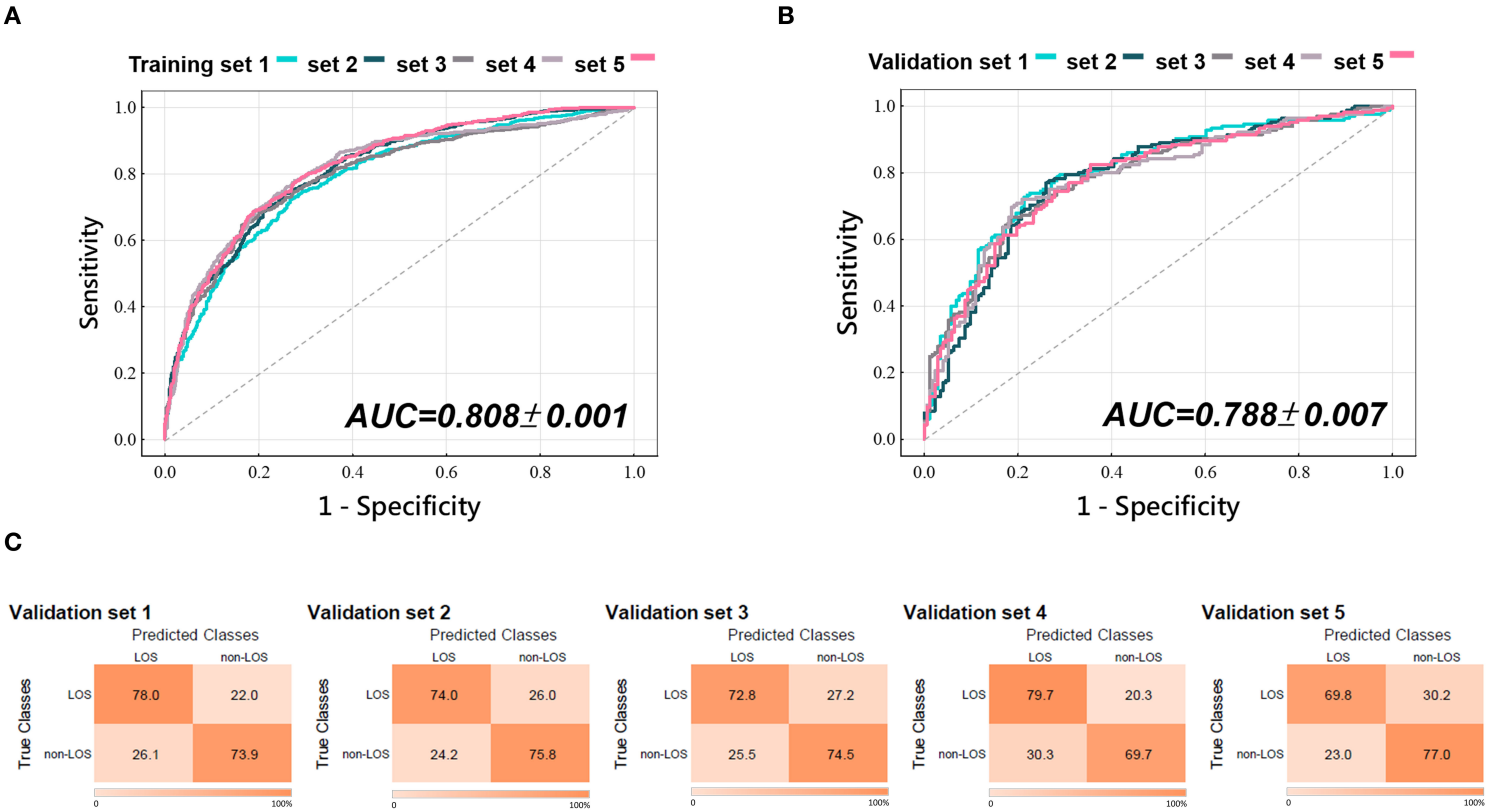
Performance of ANN models. ROC curves with AUCs of five **(A)** training and **(B)** validation sets of the ANN model for predicting AIS-associated prolonged LOS using 14 baseline parameters. AUC values are expressed as mean ± standard deviation of the five training and validation sets from the 5-fold cross-validation. **(C)** The confusion matrices with heatmaps of the five validation sets of the ANN models. The numbers in each colored box represent the percentage of instances between the true and the predicted classes obtained by the ANN models. ANN, artificial neural network; AUC, area under the curve; ROC, receiver operating characteristic.

We used 14 baseline features associated with LOS (variables with a *p*-value of < 0.05 in Table 1) as input attributes to train the ANN models to predict prolonged LOS (Figure 3). As shown in Figures 1A, B, the 2,240 patients with AIS in the cohort were randomly partitioned into training and testing sets for 5-fold cross-validation. Table 2 shows that the baseline features remained identical across the five validation sets. Following adequate training, the models that achieved the best prediction performance after 5-fold cross-validation were the ANNs containing 5, 10, 20, 25, and 30 hidden neurons. The mean AUC was 0.808 ± 0.001 for the training set (Figure 4A) and 0.788 ± 0.007 for the validation set (Figure 4B). The confusion matrices of the five validation sets were shown in Figure 4C. The mean accuracy, sensitivity, specificity, PPV, and NPV of the five validation sets were 74.5 ± 1.1%, 74.9 ± 4.0%, 74.2 ± 2.8%, 75.2 ± 1.2%, and 73.9 ± 2.5%, respectively.

**Table 2:** Comparison of the variables among five validation sets for 5-fold cross-validation.

	**Validation set 1**	**Validation set 2**	**Validation set 3**	**Validation set 4**	**Validation set 5**	***p*-value**
Number of patients	448	448	448	448	448	
Prolonged LOS, *n* (%)	220 (49.1)	220 (49.1)	220 (49.1)	220 (49.1)	221 (49.3)	1.000
Age (years)	68 (60–79)	67 (58–80)	68 (60–78)	68 (59–80)	68 (60–78)	0.877
NIHSS at admission	5 (2–9)	4 (2–10)	4 (2–12)	5 (2–10)	4 (2–10)	0.721
**Thrombolytic treatment**, ***n*** **(%)**						0.262
Non-thrombolysis	385 (85.9)	389 (86.8)	391 (87.3)	392 (87.5)	377 (84.2)	
IVT	46 (10.3)	39 (8.7)	29 (6.5)	39 (8.7)	44 (9.8)	
EVT	17 (3.8)	20 (4.5)	28 (6.3)	17 (3.8)	27 (6.0)	
**Vascular risk factors**, ***n*** **(%)**						
Atrial fibrillation	85 (19.0)	77 (17.2)	77 (17.2)	84 (18.8)	76 (17.0)	0.888
Diabetes mellitus	180 (40.2)	167 (37.3)	176 (39.3)	187 (41.7)	161 (35.9)	0.405
Hypertension	307 (68.5)	321 (71.7)	326 (72.8)	314 (70.1)	304 (67.9)	0.457
Previous stroke	80 (17.9)	70 (15.6)	57 (12.7)	71 (15.9)	70 (15.6)	0.333
**Laboratory data**						
Albumin, mg/dL	4 (3.7–4.2)	4 (3.7–4.3)	4 (3.7–4.3)	4 (3.7–4.2)	4 (3.7–4.3)	0.268
Fasting glucose, mg/dL	113 (99–146)	112 (98–147.5)	116 (100–148)	114.5 (100–159)	118 (100–151)	0.547
Hemoglobin, g/dL	13.9 (12.5–14.9)	14.1 (12.7–15.4)	14.3 (12.8–15.3)	14.3 (12.8–15.4)	14.2 (12.9–15.4)	0.054
White blood cell count, x10^3^/uL	8.4 (6.5–10.5)	8.1 (6.5–10.2)	7.9 (6.3–9.6)	8 (6.6–10)	8.2 (6.6–10.1)	0.262
Prothrombin time, sec	12.9 (12.5–13.6)	12.9 (12.4–13.6)	12.9 (12.4–13.5)	13 (12.5–13.5)	12.9 (12.4–13.5)	0.781
Cholesterol, mg/dL	186 (153–212)	184 (159.3–216.8)	184 (157.5–211)	181 (154–213)	184 (155–217)	0.816
Triglyceride, mg/dL	110 (79–153)	118 (79–161)	112 (74–173.5)	110 (77.8–152.3)	109 (75.8–162)	0.762

Continuous variables were presented as median (interquartile range, IQR), and categorical variables were expressed as counts and proportions (%). *p-*value = comparison of the variables among five validation sets. EVT, endovascular thrombectomy; IVT, intravenous thrombolysis; LOS: length of stay; NIHSS, National Institutes of Health Stroke Scale.

### 3.4. Relative significance of predictors

**Figure 5:**
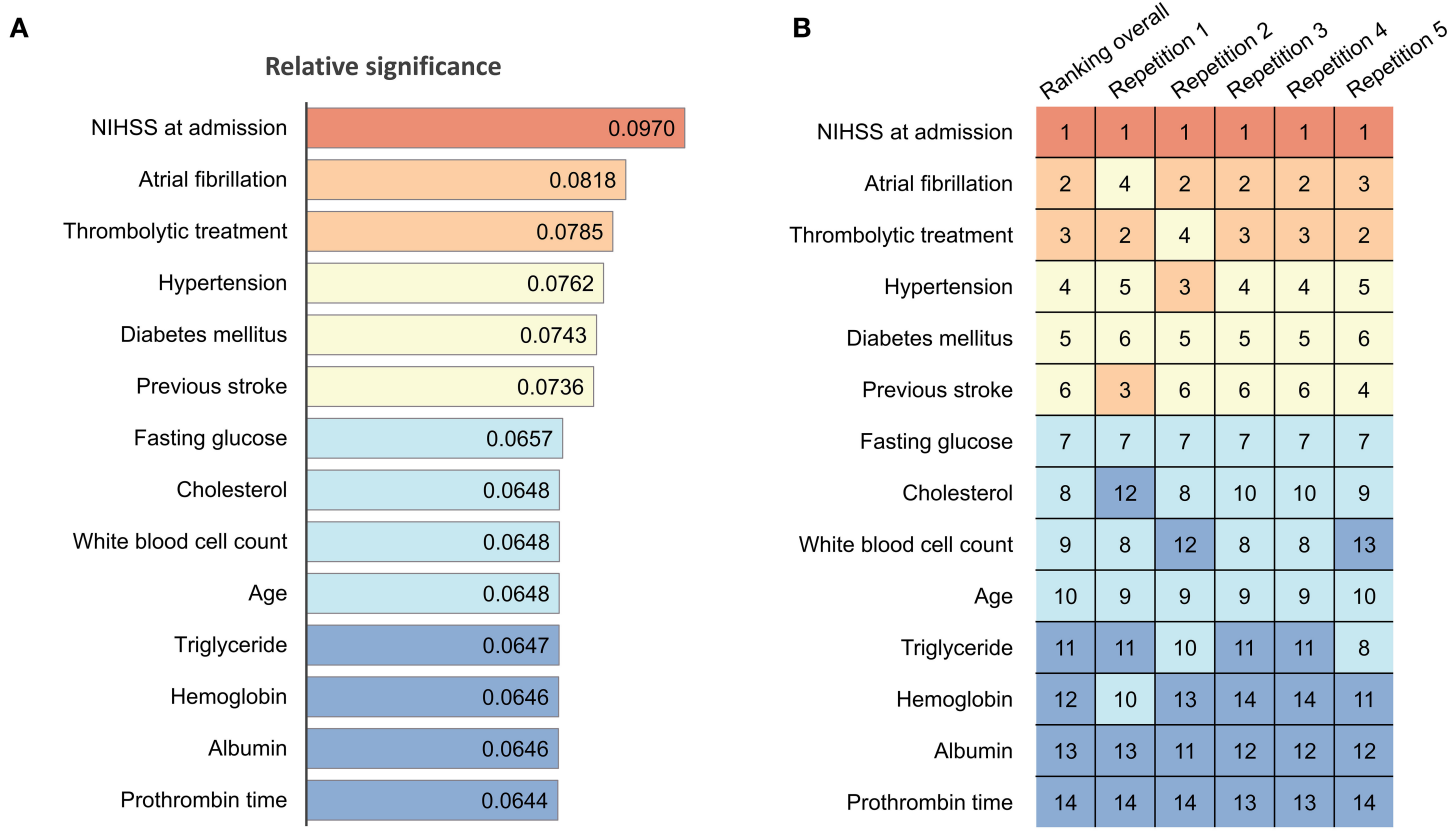
Significance and ranking of variables in ANN models. **(A)** Relative significance of each parameter in the current ANN model. The numbers in each color-coded bar indicate the mean value of the total effect of the predictors based on sensitivity analysis, with a higher value representing greater significance attributed to the models. **(B)** Heatmap and ranking of each parameter. Numbers indicate the ranking (1 = highest, 14 = lowest) of the corresponding variables. Red indicates rank 1; light brown indicates ranks 2 and 3; light yellow indicates ranks 4–6; light blue indicates ranks 7–10; and dark blue indicates ranks 11–14. ANN, artificial neural network; NIHSS, National Institutes of Health Stroke Scale.

Sensitivity analysis was performed to assess the predictive value of each parameter. The ranking of predictor significance based on the mean predictive value from each repetition of 5-fold cross-validation is shown in Figure 5. The NIHSS score at admission, Af, receiving thrombolytic therapies, history of HTN, DM, and stroke history were the strongest predictors of prolonged LOS after AIS. The relative significances of predictors of each repetition during 5-fold cross-validation are reported in Supplementary Table 1.

To assess the performance and generalizability of the prediction model for different AIS treatments, we applied the developed ANN model separately for each treatment option, namely, IVT (*n* = 197), EVT (*n* = 109), and non-thrombolytic treatments (*n* = 1,934). As shown in Table 3, the mean AUCs for predicting prolonged LOS for the non-thrombolysis, IVT, and EVT groups were 0.805 ± 0.005, 0.776 ± 0.049, and 0.696 ± 0.054, respectively. This finding indicates that the model exhibited better predictive power for the non-thrombolytic and IVT groups than for the EVT group.

**Table 3:** The performance of the current ANN model for predicting prolonged LOS of patients who received different thrombolytic treatments.

**Treatment**	**AUC**	**Accuracy**	**Precision**	**Sensitivity**	**Specificity**
Non-thrombolysis	0.805	0.733	0.768	0.690	0.778
IVT	0.776	0.720	0.686	0.855	0.573
EVT	0.696	0.588	0.540	0.943	0.267
Whole cohort	0.788	0.745	0.752	0.749	0.742

ANN, artificial neural network; AUC, area under the receiver operating characteristic curve; EVT, endovascular thrombectomy; IVT, intravenous thrombolysis; LOS, length of stay.

### 3.5. Comparison of the predicting performance of different machine learning models

To compare the performance of the ANN model to different prediction machine learning algorithms, we included the following models to predict prolonged LOS: logistic regression, support vector machine, gradient boosting tree, and random forest. All models were trained using the 14 baseline features and validated through 5-fold cross-validation. Model performance was evaluated based on the five validation sets' mean AUC, accuracy, sensitivity, specificity, PPV, and NPV of each model. We presented the various parameters used for the models in Supplementary Table 2. The results of the comparison of the five machine learning models are reported in Table 4 and showed that ANN obtained the highest AUC, accuracy, sensitivity, PPV, and NPV when compared to the other models.

**Table 4:** Comparison of the performance of machine learning models for predicting prolonged hospital length of stay.

**Model**	**AUC**	**Accuracy**	**Sensitivity**	**Specificity**	**PPV**	**NPV**
Logistic regression	0.746	0.682	0.591	**0.778**	0.736	0.647
Support vector machine	0.573	0.575	0.633	0.514	0.598	0.572
Gradient boosted tree	0.772	0.709	0.676	0.743	0.735	0.690
Random forest	0.757	0.708	0.713	0.703	0.715	0.702
ANN	**0.788**	**0.745**	**0.749**	0.742	**0.752**	**0.739**

The highest value among the five models is shown in bold. ANN, artificial neural network; AUC, area under the curve.

## 4. Discussion

### 4.1. LOS and stroke-associated economic burden

Stroke remains a leading cause of death and disability globally, and its disease and healthcare burden have increased over the past three decades (1, 2, 4). The physical and economic impacts of stroke on patients and their families have been well documented, which has increased the impact of stroke on public health (1–3). The stroke-related disability may cause prolonged LOS at hospitals and the requirement for rehabilitation, and greater LOS invariably indicates greater economic losses (7, 13). Previous studies have provided robust evidence that LOS is a major determinant of costs for patients with AIS. Prolonged hospitalization days are inevitably accompanied by an increase in medical costs, such as the consumption of beds, medications, care, and health resources. Complications associated with prolonged LOS negatively impact the prognosis and quality of life of patients with AIS and may increase the drain on medical resources and the financial burden on patients (17, 19, 20). Therefore, studies such as the present one identifying the independent predictors of LOS and developing reliable prediction methods are warranted and essential to provide data for improved decision-making related to disease management, care resource allocation, and prediction of treatment cost-effectiveness, consequently contributing positively to the individual or national healthcare management budgeting (14).

Our report showed that prolonged hospital stays are associated with worse outcomes for patients with AIS. In this study, an ANN-based model was applied to predict the risk of prolonged LOS based on the baseline characteristics of patients with AIS at admission. The ANN model achieved effective validation with an AUC of 0.788 and validation accuracy, sensitivity, specificity, PPV, and NPV of 74.5, 74.9, 74.2, 75.2, and 73.9%, respectively. These results indicate adequate discriminative power and effective prediction of the risk of prolonged LOS after AIS. Sensitivity analysis using our ANN prediction model also demonstrated that among the baseline characteristics, the NIHSS score at admission, comorbid Af, thrombolytic therapy type, HTN, DM, and history of stroke were critical predictors of prolonged LOS. These findings are consistent with a Poisson analysis-based report demonstrating that IVT and Af are significantly associated with LOS following AIS, regardless of stroke severity, age, comorbidities, or complications (39).

### 4.2. Predictive factors associated with LOS after stroke

Evidence exists that prolonged LOS is associated with poor functional outcomes and unfavorable discharge disposition in patients with AIS (17, 40). This is in concordance with current evidence that patients with a prolonged LOS exhibited a 9.3-fold higher risk of unfavorable AIS outcomes at discharge than their non-prolonged LOS counterparts. Patients with a severe disability after AIS usually need to be hospitalized for a more extended period because they may require more treatments and rehabilitation support (41–43). Although it is challenging to accurately predict LOS because of the multifactorial nature of stroke, its prediction is of paramount clinical relevance. It is generally understood that patients with initial severe motor impairment, lower functional disability levels, and a greater stroke volume might have greater LOSs and less favorable outcomes (14, 41–44). However, these correlations are not always precise because various cofactors, including patients' medical condition, stroke subtype, prehospital and in-hospital treatment, or poststroke complications, often influence the final clinical outcomes and LOS of patients with stroke (12, 13, 18, 40, 42, 45). In addition, family support and socioeconomic status affect recovery and are also the determinants of AIS-associated LOS (41, 43). Chang et al. (13) noted that stroke severity is generally associated with prolonged LOS; however, this relationship is not absolute and linear. As concluded by Bindawas et al. (46), “a short or intermediate LOS is not necessarily associated with worse outcomes, assuming adequate care is provided.”

Stroke severity is widely used for predicting prolonged LOS after AIS (13, 18, 21, 40, 42, 47). The NIHSS is a 15-item neurological examination scale with scores ranging from 0 to 42, with higher scores indicating greater stroke severity (13, 27, 40). It has been reported that for mild to moderate AIS with an NIHSS score of ≤ 15, each 1-point increase in the NIHSS score increases LOS by ~1 day, whereas for severe stroke with NIHSS scores of >15, each 1-point increase in NIHSS score causes a decrease in LOS by ~1 day, which is attributable to the high mortality rate (13). Further significance of the NIHSS score for LOS lies in evidence from retrospective AIS treatment studies, which suggested that stroke patients who benefit most from thrombolytic therapy are those with an NIHSS score of 4 to 25 on admission, whereas patients with an NIHSS score of 5 or less are more likely to be discharged home (48, 49). Patients with scores between 6 and 13 usually require inpatient rehabilitation, potentially increasing the likelihood of prolonged LOS (27, 49). In this study, our ANN model identified the NIHSS score as the strongest predictor of prolonged LOS among all variables studied. Its importance consistently ranked first in each iteration of the 5-fold cross-validation.

Consistent with our findings, specific baseline characteristics and preexisting medical conditions, including Af, HTN, DM, and previous stroke, are significant predictors of prolonged LOS in patients with AIS (12, 13, 22, 40, 45). Most of these factors associated with prolonged LOS exhibit varying prognostic relevance, thus providing some evidence-based rationale for their application as input attributes for constructing reliable prediction models. Furthermore, our improved understanding of these factors can enable the correction of modifiable risk factors, facilitate the early detection of high-risk patients, and inform disease progression, monitoring, and treatments to improve AIS outcomes. For instance, a prolonged LOS related to Af may be due to the need for anticoagulation management or cardiac evaluation. Careful monitoring of fluid and electrolyte status to prevent arrhythmic states in patients with AIS, appropriate ECG monitoring for early detection of Af, and timely anticoagulation therapy may limit the length of hospital stay (40, 50, 51). HTN and DM are modifiable risk factors of prolonged LOS in our ANN model, suggesting that controlling these factors through preventive medicine may not only reduce the risk of ischemic stroke prevalence but also reduce LOS and improve prognosis after a stroke (51). In addition, some laboratory data at the time of admission, including patients' fasting glucose and cholesterol levels, despite their relatively modest importance in our ANN model as predictors, play a role in the predictions. Providing appropriate treatment protocols for patients with AIS to optimize these profiles may further assist in reducing LOS, thereby mitigating the economic cost of AIS to individuals and society.

IVT and EVT to restore perfusion to the ischemic areas of the brain are the standard of care for patients with AIS (48), and hospital LOS is primarily affected by therapy success (21, 52). In our cohort, patients who received IVT or EVT treatment had significantly longer LOS than those who received non-thrombolytic treatment. The prolonged LOS and the effectiveness of treatment may be related to initial stroke severity or varying indications for IVT or EVT in treated patients (48). Consistent with the findings of Kasemsap et al. (39), in our study, the thrombolytic therapy type was identified as an essential predictor in our model. It is clinically relevant that our prediction model not only provides personalized estimates of risk stratification for prolonged LOS for patients receiving different therapies but also provides information that enables vital shared decision-making by patients, their families, and healthcare professionals based on the patient's condition and informed treatment preferences.

### 4.3. Comparison of contemporary prediction models for AIS-associated prolonged LOS

Predicting LOS for patients with AIS is associated with some well-documented challenges or limitations (13, 21–23, 41, 42). The 8-point Stroke subtype, Oxford Community Stroke Project classification, Age, and prestroke modified Rankin (SOAR) score based on the analysis of the data from three UK stroke registers (n = 12,355 patients with stroke) exhibited poor discrimination with an AUC of 0.61 for predicting LOS (23). The AUC was slightly higher in another model based on the International Classification of Functioning, Disability, and Health (ICF) generic set from China (50 medical centers), with a barely acceptable AUC of 0.699 (42). Similarly, Koton et al.'s prolonged LOS score based on stroke severity and type, decreased level of consciousness on admission, history of congestive heart failure, and prior Af achieved an AUC of only 0.680 in the validation cohort (22). Hung et al. (21) applied four variables, namely, NIHSS score at admission, IVT, low WBC count at admission, and age, to construct classification and regression trees for LOS prediction, and the results revealed an acceptable AUC of 0.701, with a prediction accuracy of 0.674. Other LOS prediction models included the classification of AIS subtypes as an attribute but did not yield greater discrimination power (53, 54). Considering that the accurate classification of stroke subtypes usually requires a complete investigation and elucidation of stroke etiology, we excluded the stroke subtype as a predictor in this study, which was aimed at developing a prediction model that is workable at the point of hospitalization. A recent study evaluated 30 potential predicting variables to predict LOS and found the models only explained < 25% of the LOS variance (41). The survey by Kurtz et al. (55) enrolled 16,592 stroke patients admitted to the ICU using premorbid conditions, multiple organ dysfunction parameters, and acute neurological aspects as contributing variables and compared seven different types of machine learning models to predict prolonged LOS, which yielded an AUC of 0.73 by the random forest model. Although the performance of each of the aforementioned models depends on the definition of the prolonged LOS threshold and the included population, most of the models exhibited mild–moderate predictive power. This limited predictive power may be related to the complex and multifactorial nature of AIS-associated LOS, which affects the estimate accuracy in prediction models and scoring systems. Thus, it is clinically significant that our present ANN models achieved AUCs of 0.808 and 0.788, indicating excellent discrimination power between patients with prolonged LOS and non-prolonged LOS.

Adaptive computing methods in machine learning and artificial intelligence that provide enhanced data interpretation capabilities and expand the design of efficient diagnostic and predictive tools continue to be broadly applied at the frontiers of disease management, supporting clinical decision systems (25, 29, 31, 32, 56, 57). In the literature, only a few published studies have used ANN to predict LOS in stroke patients, and neural network techniques have generally yielded good predictive capabilities (58, 59). In this study, we utilized ANN-based classifiers to develop prediction models, which generated decision signals based on weighted sums of evidence (29–31). As per available data, the relationship between predictors and outcomes is complex and multidimensional; as a machine learning tool, ANNs can analyze these complex correlations to achieve the desired effectiveness (26, 27, 29–31). Our study demonstrated the feasibility of using ANNs to predict LOS in patients with AIS. ANNs identified factors associated with prolonged LOS for establishing interventions and ensuring timely management. These factors are clinically relevant, particularly in patients with identified underlying medical conditions strongly associated with prolonged LOS. As demonstrated in this study, ANN-based algorithms can analyze large-scale and non-linear data for developing new integrated methods, including different informatics features to predict LOS in patients with AIS. This enables healthcare stakeholders to use patient information obtained at admission to estimate the risk of prolonged LOS, share clinical and cost decisions, provide treatment options during hospitalization, and arrange referral pathways after discharge. For patients at high risk of prolonged LOS, hospital management can adjust strategies accordingly to improve the allocation of health resources, and insurance providers can develop reimbursement policies. This prediction model can help governments improve resource allocation, project cost-effectiveness, and contribute to individual and national health insurance budgets.

### 4.4. Limitations

Our study has some limitations. First, this was a single-center study with a moderate sample size (*n* = 2,240) and without an external validation dataset. Therefore, further generalization of the developed model requires a large multicenter cohort with extensively varied characteristics that represent the disease population to be conducted, which can also validate our results. Second, this observational study may have been affected by unmeasured confounding variables. Similar to most contemporary models, this study did not consider the effects of differences related to socioeconomic status and medical service heterogeneity on LOS (41–43, 60), which may affect the model's predictive power. However, in Taiwan, the high coverage provided by the national health insurance system (61) reduces the relative inconsistency of medical care services, making our model more clinically applicable. Third, our estimates were generated at the time of admission and did not include poststroke complications or stroke subtypes that caused prolonged LOS during hospitalization (12, 18, 40), limiting model validity. Fourth, our study considered LOS in a single center during the acute phase, excluding the days a patient with stroke may have spent in rehabilitation or in a long-term care facility after stabilization of stroke. This may limit the predictive accuracy of the model.

## 5. Conclusion

Our study identified crucial predictive factors and developed ANN models that accurately and effectively predicted prolonged LOS in patients with AIS based on clinical parameters obtained at admission. NIHSS, Af, thrombolytic therapies, HTN, DM, and previous stroke history were the strongest predictors of prolonged LOS after AIS. Prolonged LOS is associated with poor functional outcomes at discharge in patients with AIS. This machine learning-based model contributes to further understanding to improve healthcare management and resource allocation in the stroke unit related to LOS. Providing appropriate treatment to optimize modifiable risk factors of prolonged LOS may assist in reducing LOS, thereby mitigating the economic cost of AIS to individuals and society. The models reported in this study are clinically applicable and workable and can be used to inform decision-making and formulate individualized stroke inpatient care plans.

## Data availability statement

The data analyzed in this study was obtained from the Taiwan Stroke Registry (TSR; http://taiwanstrokeregistry.org/TSR/), the following licenses/restrictions apply: The datasets for the current research were used under a license and so are not publicly available. Requests to access these datasets should be directed to the Taiwan Stroke Registry, taiwanstrokeregistry@gmail.com.

## Ethics statement

The studies involving human participants were reviewed and approved by Joint Institutional Review Board of Taipei Medical University. Written informed consent for participation was not required for this study in accordance with the national legislation and the institutional requirements.

## Author contributions

C-CC contributed to the study conception and design and the provision of resources, and administrative oversight. C-CY, C-TH, LC, and C-CC contributed to the data acquisition and analysis. OB, LC, C-TH, and C-CC contributed to the data interpretation. C-CY, OB, J-HC, C-TH, Y-TH, and C-CC contributed to the manuscript writing. All authors contributed to the study and approved the final version of the manuscript.

## References

[1] Feigin VL Stark BA Johnson CO Roth GA Bisignano C Abady GG. Global, regional, and national burden of stroke and its risk factors, 1990–2019: a systematic analysis for the Global Burden of Disease Study 2019. Lancet Neurol. (2021) 20:795–820. 10.1016/S1474-4422(21)00252-0 34487721 PMC8443449

[2] Katan M Luft A. Global burden of stroke. Semin Neurol. (2018) 38:208–11. 10.1055/s-0038-1649503 29791947

[3] Rajsic S Gothe H Borba HH Sroczynski G Vujicic J Toell T. Economic burden of stroke: a systematic review on post-stroke care. Eur J Health Econ. (2019) 20:107–34. 10.1007/s10198-018-0984-0 29909569

[4] Luengo-Fernandez R Violato M Candio P Leal J. Economic burden of stroke across Europe: A population-based cost analysis. Eur Stroke J. (2020) 5:17–25. 10.1177/2396987319883160 32232166 PMC7092742

[5] Wafa HA Wolfe CDA Emmett E Roth GA Johnson CO Wang Y. Burden of stroke in Europe. Stroke. (2020) 51:2418–27. 10.1161/STROKEAHA.120.029606 32646325 PMC7382540

[6] Ovbiagele B Goldstein LB Higashida RT Howard VJ Johnston SC Khavjou OA. Forecasting the future of stroke in the United States: a policy statement from the American Heart Association and American Stroke Association. Stroke. (2013) 44:2361–75. 10.1161/STR.0b013e31829734f2 23697546

[7] Teo K Slark J. A systematic review of studies investigating the care of stroke survivors in long-term care facilities. Disabil Rehabil. (2016) 38:715–23. 10.3109/09638288.2015.1059496 26104106

[8] Su M Pan D Zhao Y Chen C Wang X Lu W. The direct and indirect effects of length of hospital stay on the costs of inpatients with stroke in Ningxia, China, between 2015 and 2020: a retrospective study using quantile regression and structural equation models. Front Public Health. (2022) 10:881273. 10.3389/fpubh.2022.881273 36033765 PMC9415100

[9] Chen C Zhu B Chen Y Yang T Li F. Factors influencing the hospitalization cost for stroke patients in J district, Shanghai. Zhong Nan Da Xue Xue Bao Yi Xue Ban. (2022) 47:628–38. 10.11817/j.issn.1672-7347.2022.210429 35753733 PMC10929916

[10] Lu Y Sun W Shen Z Sun W Liu R Li F. Regional differences in hospital costs of acute ischemic stroke in china: analysis of data from the chinese acute ischemic stroke treatment outcome registry. Front Public Health. (2021) 9:783242. 10.3389/fpubh.2021.783242 34957035 PMC8702643

[11] Khan SU Khan MZ Khan MU Khan MS Mamas MA Rashid M. Clinical and economic burden of stroke among young, midlife, and older adults in the United States, 2002–2017. Mayo Clin Proc Innov Qual Outcomes. (2021) 5:431–41. 10.1016/j.mayocpiqo.2021.01.015 33997639 PMC8105541

[12] Huang YC Hu CJ Lee TH Yang JT Weng HH Lin LC. The impact factors on the cost and length of stay among acute ischemic stroke. J Stroke Cerebrovasc Dis. (2013) 22:e152–8. 10.1016/j.jstrokecerebrovasdis.2012.10.014 23253537

[13] Chang K-C Tseng M-C Weng H-H Lin Y-H Liou C-W Tan T-Y. Prediction of length of stay of first-ever ischemic stroke. Stroke. (2002) 33:2670–4. 10.1161/01.STR.0000034396.68980.39 12411659

[14] McClure JA Salter K Meyer M Foley N Kruger H Teasell R. Predicting length of stay in patients admitted to stroke rehabilitation with high levels of functional independence. Disabil Rehabil. (2011) 33:2356–61. 10.3109/09638288.2011.572225 21504345

[15] Baek H Cho M Kim S Hwang H Song M Yoo S. Analysis of length of hospital stay using electronic health records: a statistical and data mining approach. PLoS ONE. (2018) 13:e0195901. 10.1371/journal.pone.0195901 29652932 PMC5898738

[16] Song J Chen C Zhao S Zhou L Chen H. Trading quality for quantity? Evidence from patient level data in China. PLoS ONE. (2021) 16:e0257127. 10.1371/journal.pone.0257127 34529680 PMC8445449

[17] Lin KH Lin HJ Yeh PS. Determinants of prolonged length of hospital stay in patients with severe acute ischemic stroke. J Clin Med. (2022) 11:3457. 10.3390/jcm11123457 35743530 PMC9225000

[18] George AJ Boehme AK Siegler JE Monlezun D Fowler BD Shaban A. Hospital-acquired infection underlies poor functional outcome in patients with prolonged length of stay. ISRN Stroke. (2013) 2013:312348. 10.1155/2013/312348 24377056 PMC3873143

[19] Paolucci S Iosa M Coiro P Venturiero V Savo A De Angelis D. Post-stroke depression increases disability more than 15% in ischemic stroke survivors: a case-control study. Front Neurol. (2019) 10:926. 10.3389/fneur.2019.00926 31507525 PMC6718567

[20] Sugawara N Metoki N Hagii J Saito S Shiroto H Tomita T. Effect of depressive symptoms on the length of hospital stay among patients hospitalized for acute stroke in Japan. Neuropsychiatr Dis Treat. (2015) 11:2551–6. 10.2147/NDT.S91303 26491334 PMC4599635

[21] Hung L-C Hu Y-H Sung S-F. Exploring the impact of intravenous thrombolysis on length of stay for acute ischemic stroke: a retrospective cohort study. BMC Health Serv Res. (2015) 15:404. 10.1186/s12913-015-1080-0 26399930 PMC4580364

[22] Koton S Bornstein NM Tsabari R Tanne D. Derivation and validation of the prolonged length of stay score in acute stroke patients. Neurology. (2010) 74:1511–6. 10.1212/WNL.0b013e3181dd4dc5 20458067

[23] Myint PK Clark AB Kwok CS Davis J Durairaj R Dixit AK. The SOAR (stroke subtype, oxford community stroke project classification, age, prestroke modified Rankin) score strongly predicts early outcomes in acute stroke. Int J Stroke. (2014) 9:278–83. 10.1111/ijs.12088 23834262

[24] Suzuki S Yamashita T Sakama T Arita T Yagi N Otsuka T. comparison of risk models for mortality and cardiovascular events between machine learning and conventional logistic regression analysis. PLoS ONE. (2019) 14:e0221911. 10.1371/journal.pone.0221911 31499517 PMC6733605

[25] Chou S-Y Bamodu OA Chiu W-T Hong C-T Chan L Chung C-C. Artificial neural network-boosted cardiac arrest survival post-resuscitation in-hospital (CASPRI) score accurately predicts outcome in cardiac arrest patients treated with targeted temperature management. Sci Rep. (2022) 12:7254. 10.1038/s41598-022-11201-z 35508580 PMC9068683

[26] Kuan YC Hong CT Chen PC Liu WT Chung CC. Logistic regression and artificial neural network-based simple predicting models for obstructive sleep apnea by age, sex, and body mass index. Math Biosci Eng. (2022) 19:11409–21. 10.3934/mbe.2022532 36124597

[27] Chung CC Bamodu OA Hong CT Chan L Chiu HW. Application of machine learning-based models to boost the predictive power of the SPAN index. Int J Neurosci. (2021) 133:26–36. 10.1080/00207454.2021.1881092 33499706

[28] Arkin FS Aras G Dogu E. Comparison of artificial neural networks and logistic regression for 30-days survival prediction of cancer patients. Acta Inform Med. (2020) 28:108–13. 10.5455/aim.2020.28.108-113 32742062 PMC7382770

[29] Amato F López A Peña-Méndez EM Vanhara P Hampl A Havel J. Artificial neural networks in medical diagnosis. J Appl Biomed. (2013) 11:47–58. 10.2478/v10136-012-0031-x25058735

[30] Zou J Han Y So SS. Overview of artificial neural networks. Methods Mol Biol. (2008) 458:15–23. 10.1007/978-1-60327-101-1_2 19065803

[31] Shahid N Rappon T Berta W. Applications of artificial neural networks in health care organizational decision-making: a scoping review. PLoS ONE. (2019) 14:e0212356. 10.1371/journal.pone.0212356 30779785 PMC6380578

[32] Chung C-C Chan L Bamodu OA Hong C-T Chiu H-W. Artificial neural network based prediction of post-thrombolysis intracerebral hemorrhage and death. Sci Rep. (2020) 10:20501. 10.1038/s41598-020-77546-5 33239681 PMC7689530

[33] Hsieh FI Lien LM Chen ST Bai CH Sun MC Tseng HP. Get with the guidelines-stroke performance indicators: surveillance of stroke care in the Taiwan stroke registry: get with the guidelines-stroke in Taiwan. Circulation. (2010) 122:1116–23. 10.1161/CIRCULATIONAHA.110.936526 20805428

[34] Lee SY Lee SH Tan JHH Foo HSL Phan PH Kow AWC. Factors associated with prolonged length of stay for elective hepatobiliary and neurosurgery patients: a retrospective medical record review. BMC Health Serv Res. (2018) 18:5. 10.1186/s12913-017-2817-8 29304787 PMC5755148

[35] Lee AH Fung WK Fu B. Analyzing hospital length of stay: mean or median regression? Med Care. (2003) 41:681–6. 10.1097/01.MLR.0000062550.23101.6F 12719692

[36] Nawi NM Ransing MR Ransing RS. “An improved learning algorithm based on the broyden-fletcher-goldfarb-shanno (BFGS) method for back propagation neural networks,” In: *Sixth International Conference on Intelligent Systems Design and Applications*. (2006). p. 16–18. 10.1109/ISDA.2006.95

[37] Sobol′ IM. Global sensitivity indices for nonlinear mathematical models and their Monte Carlo estimates. Math Comput Simulat. (2001) 55:271–80. 10.1016/S0378-4754(00)00270-6

[38] Chung CC Chiu WT Huang YH Chan L Hong CT Chiu HW. Identifying prognostic factors and developing accurate outcome predictions for in-hospital cardiac arrest by using artificial neural networks. J Neurol Sci. (2021) 425:117445. 10.1016/j.jns.2021.117445 33878655

[39] Kasemsap N Vorasoot N Kongbunkiat K Peansukwech U Tiamkao S Sawanyawisuth K. Impact of intravenous thrombolysis on length of hospital stay in cases of acute ischemic stroke. Neuropsychiatr Dis Treat. (2018) 14:259–64. 10.2147/NDT.S151836 29386899 PMC5767097

[40] Mohamed W Bhattacharya P Shankar L Chaturvedi S Madhavan R. Which comorbidities and complications predict ischemic stroke recovery and length of stay? Neurologist. (2015) 20:27–32. 10.1097/NRL.0000000000000040 26280287

[41] García-Rudolph A Cegarra B Opisso E Tormos JM Bernabeu M Saurí J. Predicting length of stay in patients admitted to stroke rehabilitation with severe and moderate levels of functional impairments. Medicine. (2020) 99:e22423. 10.1097/MD.0000000000022423 33120737 PMC7581132

[42] Zhang X Qiu H Liu S Li J Zhou M. Prediction of prolonged length of stay for stroke patients on admission for inpatient rehabilitation based on the international classification of functioning, disability, and health (ICF) generic set: a study from 50 centers in China. Med Sci Monit Int Med J Exp Clin Res. (2020) 26:e918811. 10.12659/MSM.918811 31901931 PMC6977619

[43] Tan WS Heng BH Chua KS Chan KF. Factors predicting inpatient rehabilitation length of stay of acute stroke patients in Singapore. Arch Phys Med Rehabil. (2009) 90:1202–7. 10.1016/j.apmr.2009.01.027 19577034

[44] Maddox JM MacWalter RS McMahon AD. Relationship of volume of lesion to length of hospital stay and outcome at one year in stroke patients. Scott Med J. (2001) 46:178–83. 10.1177/003693300104600609 11852633

[45] Jørgensen HS Nakayama H Raaschou HO Olsen TS. Acute stroke care and rehabilitation: an analysis of the direct cost and its clinical and social determinants. Copenhagen Stroke Study Stroke. (1997) 28:1138–41. 10.1161/01.STR.28.6.1138 9183339

[46] Bindawas SM Vennu V Mawajdeh H Alhaidary HM Moftah E. Length of stay and functional outcomes among patients with stroke discharged from an inpatient rehabilitation facility in Saudi Arabia. Med Sci Monit Int Med J Exp Clin Res. (2018) 24:207–14. 10.12659/MSM.907452 29321468 PMC5772339

[47] Ntaios G Papavasileiou V Michel P Tatlisumak T Strbian D. Predicting functional outcome and symptomatic intracranial hemorrhage in patients with acute ischemic stroke: a glimpse into the crystal ball? Stroke. (2015) 46:899–908. 10.1161/STROKEAHA.114.003665 25657189

[48] Powers WJ Rabinstein AA Ackerson T Adeoye OM Bambakidis NC Becker K. 2018 guidelines for the early management of patients with acute ischemic stroke: a guideline for healthcare professionals from the American Heart Association/American Stroke Association. Stroke. (2018) 49:e46–e110. 10.1161/STR.0000000000000158 29367334

[49] Schlegel D Kolb SJ Luciano JM Tovar JM Cucchiara BL Liebeskind DS. Utility of the NIH Stroke Scale as a predictor of hospital disposition. Stroke. (2003) 34:134–7. 10.1161/01.STR.0000048217.44714.02 12511764

[50] Seiffge DJ Werring DJ Paciaroni M Dawson J Warach S Milling TJ. Timing of anticoagulation after recent ischaemic stroke in patients with atrial fibrillation. Lancet Neurol. (2019) 18:117–26. 10.1016/S1474-4422(18)30356-9 30415934 PMC6524642

[51] Kleindorfer DO Towfighi A Chaturvedi S Cockroft KM Gutierrez J Lombardi-Hill D. Guideline for the prevention of stroke in patients with stroke and transient ischemic attack: a guideline from the American Heart Association/American Stroke Association. Stroke. (2021) 52:e364–467. 10.1161/STR.0000000000000375 34024117

[52] Minaeian A Patel A Essa B Goddeau RP Moonis M Henninger N. Emergency department length of stay and outcome after ischemic stroke. J Stroke Cerebrovasc Dis. (2017) 26:2167–73. 10.1016/j.jstrokecerebrovasdis.2017.04.040 28551289 PMC5600670

[53] Appelros P. Prediction of length of stay for stroke patients. Acta Neurol Scand. (2007) 116:15–9. 10.1111/j.1600-0404.2006.00756.x 17587250

[54] Al Taleb AR Hoque M Hasanat A Khan MB. Application of data mining techniques to predict length of stay of stroke patients. Int Conf Inf Health Technol. (2017) 1–5. 10.1109/ICIHT.2017.7899004

[55] Kurtz P Peres IT Soares M Salluh JIF Bozza FA. Hospital length of stay and 30-day mortality prediction in stroke: a machine learning analysis of 17,000 ICU admissions in Brazil. Neurocrit Care. (2022) 37(Suppl. 2):313–21. 10.1007/s12028-022-01486-3 35381967

[56] Davenport T Kalakota R. The potential for artificial intelligence in healthcare. Future Healthcare J. (2019) 6:94–8. 10.7861/futurehosp.6-2-94 31363513 PMC6616181

[57] Yu K-H Beam AL Kohane IS. Artificial intelligence in healthcare. Nat Biomed Eng. (2018) 2:719–31. 10.1038/s41551-018-0305-z 31015651

[58] Bacchi S Oakden-Rayner L Menon DK Jannes J Kleinig T Koblar S. Stroke prognostication for discharge planning with machine learning: a derivation study. J Clin Neurosci. (2020) 79:100–3. 10.1016/j.jocn.2020.07.046 33070874

[59] Neto C Brito M Peixoto H Lopes V Abelha A Machado J. Prediction of Length of Stay for Stroke Patients Using Artificial Neural Networks. Trends and Innovations in Information Systems and Technologies. Cham: Springer International Publishing (2020). 10.1007/978-3-030-45688-7_2233070874

[60] Tørnes M McLernon D Bachmann M Musgrave S Warburton EA Potter JF. Does service heterogeneity have an impact on acute hospital length of stay in stroke? A UK-based multicentre prospective cohort study. BMJ Open. (2019) 9:e024506. 10.1136/bmjopen-2018-024506 30948571 PMC6500188

[61] Lin L-Y Warren-Gash C Smeeth L Chen P-C. Data resource profile: the National Health Insurance Research Database (NHIRD). Epidemiol Health. (2018) 40:e2018062. 10.4178/epih.e2018062 30727703 PMC6367203

