# Supplementary material for: Risk factor identification and prediction models for prolonged length of stay in hospital after acute ischemic stroke using artificial neural networks
Source: Front Neurol. 2023 Feb 9;14:1085178. doi: 10.3389/fneur.2023.1085178 (PMC9947790; doi:10.3389/fneur.2023.1085178)
Supplement: Supplementary file 1 [file Data_Sheet_1.PDF]

Supplementary Table 1. Relative significance of predictors in each repetition of cross-validation.

Variables	Relative significance				
	Repetition 1	Repetition 2	Repetition 3	Repetition 4	Repetition 5
NIHSS at admission	0.0899	0.0981	0.0993	0.1038	0.0937
Atrial fibrillation	0.0721	0.0875	0.0782	0.0891	0.0823
Thrombolytic treatment	0.0755	0.0752	0.0738	0.0856	0.0826
Hypertension	0.0719	0.0812	0.0729	0.0777	0.0771
Diabetes mellitus	0.0711	0.0726	0.0760	0.0776	0.0744
Previous stroke	0.0739	0.0711	0.0728	0.0716	0.0785
Fasting glucose	0.0695	0.0647	0.0660	0.0629	0.0653
Cholesterol	0.0680	0.0643	0.0662	0.0617	0.0640
White blood cell count	0.0684	0.0642	0.0660	0.0622	0.0635
Age	0.0681	0.0643	0.0656	0.0620	0.0640
Triglyceride	0.0680	0.0643	0.0658	0.0617	0.0640
Hemoglobin	0.0680	0.0641	0.0661	0.0612	0.0638
Albumin	0.0678	0.0642	0.0657	0.0616	0.0636
Prothrombin time	0.0677	0.0641	0.0656	0.0614	0.0633

NIHSS, National Institutes of Health Stroke Scale
