# Supplementary material for: Risk factor identification and prediction models for prolonged length of stay in hospital after acute ischemic stroke using artificial neural networks
Source: Front Neurol. 2023 Feb 9;14:1085178. doi: 10.3389/fneur.2023.1085178 (PMC9947790; doi:10.3389/fneur.2023.1085178)
Supplement: Supplementary file 2 [file Data_Sheet_2.PDF]

Supplementary Table 2. Summary of the parameters of machine learning models in the current study

Model	Parameters
Logistic regression	Parametrization: sigma-restricted<br>Model building: all effects
Support vector machine	Kernel type: radial basis function<br>Degree: 3
Gradient boosted tree	The maximum number of nodes for each tree: 3<br>The minimum number of cases: 50<br>The maximum number of levels: 10
Random forest	Number of trees: 100<br>Number of predictors: 4<br>Random test data proportion: 0.3<br>Subsample proportion: 0.5<br>The minimum number of cases: 50<br>The maximum number of levels: 10<br>The maximum number of nodes: 100
ANN	One input, one hidden, and one output layers<br>The hidden layer: 1–50 neurons; activation, exponential or the hyperbolic tangent function<br>The output layer: 2 neurons; activation is the sigmoid function<br>Error function: the cross-entropy error function

ANN: artificial neural network.
